# Do we need to go further to train healthcare providers in the targeted regions for malaria elimination in Myanmar? A mixed-methods study

**DOI:** 10.1186/s41182-020-00196-w

**Published:** 2020-02-21

**Authors:** Zar Ni Min Hein, Thae Maung Maung, Poe Poe Aung, Nwe Oo Mon, Wai Wai Han, Tin Oo, Nay Yi Yi Linn, Aung Thi, Khin Thet Wai

**Affiliations:** 1 Department of Medical Research, Ministry of Health and Sports, Yangon, Myanmar; 2 Duke Global Health Institute Myanmar Program, Yangon, Myanmar; 3 National Malaria Control Program, Department of Public Health, Ministry of Health and Sports, Nay Pyi Taw, Myanmar

**Keywords:** Malaria elimination, Training, Health staff, Myanmar, SORT IT, Knowledge, Perceptions

## Abstract

**Background:**

The National Malaria Control Programme (NMCP) in Myanmar trained health staff at the township level starting in mid-2016 in order to achieve the *Plasmodium falciparum* malaria elimination target by 2020. This study aimed to evaluate the knowledge and perception of Basic Health Staff (BHS) and Vector-borne Diseases Control (VBDC) teams exposed to a short training course on malaria elimination in six targeted townships which included two conflict-affected townships between 2016 and 2017.

**Methods:**

This was a cross-sectional mixed-methods study using quantitative and qualitative data extracted from one survey database conducted between October 2018 and March 2019. Modified Poisson regression analysis was performed to ascertain the determinants of low knowledge scores after the training programme.

**Results:**

Altogether, 544 trained frontline health workers involved in malaria elimination at the time of the survey were recruited and 56% (302/544) were stationed at sub-Rural Health Centers. More than half of the respondents had correct knowledge of malaria case categories although relapse and recrudescent cases (39% and 37% respectively) were less well known. Over two-thirds of respondents could mention those eligible for malaria testing. Less than 30% knew the foci classification. The overall knowledge scores ranged from 10 to 31. The significant predictors of low level of knowledge [the cut-off point was set at the median value of 21 (IQR 12–30)] in multivariate analysis were the younger age group (18–29 years) and health staff who had attended malaria elimination training in 2017, [(APR = 1.6, 95% CI 1.2–2.2)]; and (APR = 1.5, 95%CI 1.2–1.8)]. Qualitative data from 10 key informants identified perceived challenges in conflict-affected areas as well as in areas of high population mobility with further implications for case surveillance. In addition, the low level of education of community members was noted as one of the barriers that hampered public readiness in the elimination scenario.

**Conclusion:**

A significant impact on knowledge improvement after the training programme was not visible especially for correct notification of malaria cases and treatment according to National Malaria Elimination Guidelines. Regular monitoring and continuing guidance by the higher level management is critical to support the field staff.

## Background

Malaria elimination is defined as the interruption of indigenous transmission of a specified malaria parasite species in a well-defined geographic area as a result of concerted elimination efforts [1]. In 22 countries of Asia and the Pacific region including Myanmar, approximately 2.1 billion people (80% of the total population) were at risk of getting malaria in 2015 [2]. The WHO Global Technical Strategy for Malaria (2016-2030) aims at a 90% reduction of cases by 2030 compared to 2015 [3]. Malaria morbidity was reduced by 72% in 2016 in Myanmar compared to 2012 and there was a 95% reduction in malaria deaths within a 4-year period. However, the problem of artemisinin resistance has emerged as one of the impediments to elimination [4–7]. Moreover, conflict-affected settings and areas with high population mobility have enhanced the programmatic challenges of moving towards elimination [8–10].

The National Malaria Control Programme (NMCP) in Myanmar has set its elimination goals in 2016 to cover six out of 14 states/regions in Myanmar and a Union Territory. Strengthening the surveillance system remains one of the important core interventions in malaria elimination [4]. The NMCP staff have worked closely with Basic Health Staff (BHS) to provide training at the township level starting from mid-2016 in order to achieve the *Plasmodium falciparum* malaria elimination target by 2020 [11]. At the operational level in countries aiming for malaria elimination, malaria control programmes are faced with challenges in health system readiness that include three domains: staff and guidelines, diagnostics, and medicines and commodities [12]. The National Malaria Elimination Guidelines are fundamental in building strong partnerships among well-trained frontline workers as the key investigators for surveillance activities at the implementation sites. Furthermore, improved capacity of staff may assist thorough analyses of determinants of malaria transmission in low endemic settings including Myanmar [1–3, 12].

The role of local health staff in malaria elimination in Myanmar has become increasingly important for the identification of cases and for adequate treatment to mitigate subsequent transmission. In this regard, skilful health workers are essential at all levels of the healthcare infrastructure: this includes subnational level management, township level programme management, and field supervision and management. Whether the current training programme in targeted townships can adequately serve the purpose of malaria elimination by helping to overcome the challenges encountered by field-level staff still needs to be explored.

A PubMed literature search revealed only two studies from the African Region and one study from the Asia and the Pacific Region that have recognized the importance of staff capacity and attitudes together with multi-sectoral collaboration in the malaria elimination scenario [13–15].

This study, therefore, aimed to evaluate the knowledge as well as the perception of BHS and members of Vector-borne Diseases Control (VBDC) teams following a short training course on malaria elimination in six targeted townships between 2016 and 2017. Specific objectives were to describe: (i) the social, demographic, and programme-specific characteristics of respondents; (ii) the knowledge of health staff responsible for field supervision related to malaria elimination and its associated factors; and (iii) perceived challenges of township level health staff responsible for programme management related to capacity strengthening interventions in six targeted townships for malaria elimination. This information could further influence and strengthen the readiness of such staff to engage in programme implementation strategies for elimination in targeted settings as early as 2020.

## Results

### The quantitative strand

Table 1 presents the social, demographic, and programme-specific characteristics**.** The median age of respondents was 28 years (IQR 23–38) (range 19–59 years). About 88% (479/544) had attained up to university level education. Around 15% (79/544) were responsible for township level programme management (THN, HA, LHV) and the rest were involved in field supervision (MW, PHS, VBDC staff). Nearly one-third of the respondents were recruited from two conflict-affected townships where the elimination training was initiated in 2016 and 56% (302/544) belonged to a sub-center as their duty station. In contrast to conflict-affected townships, preliminary arrangements for the training schedule were convenient without any postponement in unaffected sites. Moreover, the trained frontline health workers found no difficulty in carrying out the malaria elimination activities in study sites without any conflict.

**Table 1:** Background characteristics of BHS and VBDC teams at six targeted townships for malaria elimination, Myanmar, 2017 to 2018 (*n* = 544)

Characteristic	No.	(%)
Age group in years* (*n* = 542)		
18–29	289	(54)
30–39	137	(25)
40–49	72	(13)
≥ 50	44	(8)
Education		
Middle school/high school	65	(12)
University/graduate	479	(88)
Designation		
THN/THA	5	(1)
HA	41	(7)
LHV	33	(6)
MW	248	(46)
PHS	206	(38)
VBDC staff	11	(2)
Duty station		
Township hospital	20	(4)
Station hospital	21	(4)
MCH	41	(7)
RHC	160	(29)
Sub-centre	302	(56)
Initiation of elimination training in townships		
2016	179	(33)
2017	365	(67)

*THN* Township Health Nurse, *THA* Township Health Assistant, *HA* Health Assistant, *LHV* Lady Health Visitor, *MW* Midwife, *PHS* Public Health Supervisor, *VBDC* Vector-borne Diseases Control staff, *SHU* Station Health Unit, *MCH* Maternal and Child Health Centre, *RHC* Rural Health Centre

*Age information of 2 participants was missing

As for critical knowledge elements (see Table 2), the respondents were more likely to correctly define malaria cases than malaria elimination (45% vs. 25%). However, over 95% of BHS knew about surveillance and the provision of health education as malaria elimination activities. Even though more than half of the respondents could express correct knowledge of malaria case categories, they were less likely to mention relapse and recrudescent cases (39% and 37% respectively) imperative for case-finding activities. Over two-thirds of BHS could mention the eligible persons for malaria testing except for those with anemia of unknown cause (44%). In contrast, less than 30% knew the foci classification.

**Table 2:** Knowledge about malaria elimination at six targeted townships for malaria elimination, Myanmar, 2017 to 2018

Knowledge^†^	*n* = 544	
	No.	(%)
Knowledge of definition of malaria elimination	134	(25)
Knowledge of definition of malaria case	242	(45)
Knowledge on activities for malaria elimination		
Finding malaria patients, check blood test and effective treatment	541	(99)
Surveillance	524	(96)
Health education about malaria	535	(98)
Vector control activities	435	(80)
Category of malaria cases		
Indigenous case	332	(61)
Introduced case	252	(46)
Imported case	296	(54)
Relapse case	216	(39)
Induced case	338	(62)
Recrudescent case	203	(37)
Those who are required to test for malaria		
Patients with fever, malaise and chills	514	(94)
All febrile patients from malaria foci, especially during the transmission season	527	(97)
People with a history of malaria/having visited a malaria endemic area in the past 3 years and any increase in body temperature	498	(91)
People with anaemia of unknown cause	241	(44)
Patients with hepatomegaly or splenomegaly (or both)	420	(77)
Recipients of donated blood who have fever during three months after the transfusion	477	(88)
In low transmission intensity setting or transmission is assumed to be interrupted, people surrounding the index case(s) should be tested regardless of symptoms	383	(70)
Types of foci in foci investigation		
Active foci	161	(30)
Residual non-active foci	143	(26)
Clear foci	145	(27)

^**†**^Correct responses

According to Table 3, the large majority of BHS (96%) recognized that universal access to and use of treated bed nets was one of the core interventions for malaria vector control. Of five key messages aimed at the community to engage them in the prevention and control of malaria, only 74% of respondents were able to state ‘to clean the environment’. The overall knowledge scores ranged from 10 to 31 with a median value of 21 (IQR 12–30).

**Table 3:** Knowledge about vector control interventions and awareness of key malaria messages at six targeted townships for malaria elimination, Myanmar, 2017 to 2018

Knowledge^†^	*n* = 544	
	No.	(%)
Core interventions for malaria vector control		
Universal access to and use of ITN/LLINs	524	(96)
Clean the environment	178	(33)
Universal access to IRS for population at risk for malaria	299	(55)
Aware of key messages to be conveyed		
Systematic use of LLINs	531	(98)
Check blood test within 24 h from the start of fever	498	(91)
Taking full course treatment	530	(97)
Cleaning the environment	402	(74)
Reporting if malaria epidemic occurred and preventive measure in malaria-free area	505	(93)
Removal of the trash properly	221	(41)

^**†**^Correct responses

*ITN* Insecticide-treated nets, *LLINS* Long-lasting insecticidal nets, *IRS* Indoor residual spraying

Table 4 presents the logistic regression analysis assessing the factors associated with the knowledge level of health staff in six targeted townships. When adjusted for other variables, those in the younger age group (18–29 years) and age group (30–39 years) had a significant association with low knowledge scores [(APR = 1.6, 95%CI 1.2–2.2)] and [(APR = 1.4, 95%CI 1.0–1.9)] respectively. There was a significant association between low low education status and low knowledge scores [(APR = 1.3, 95%CI 1.0–1.7)]. Surprisingly, health staff who had attended malaria elimination training in 2017 had a significantly low level of knowledge compared with the reference category [(APR = 1.5, 95% CI 1.2–1.8)] when controlling for other variables.

**Table 4:** Factors associated with knowledge of malaria elimination among the health staff and VBDC teams at six targeted townships for malaria elimination, Myanmar, 2017 to 2018 (*n* = 544)

Characteristics	Total (*n*)	Low knowledge		Unadjusted PR (95% CI)	*p* value	Adjusted PR (95% CI)	*p* value
		*N*	(%)				
Age group*	542						
≥ 40 years	116	46	(40)	1		1	
18–29 years	289	166	(57)	1.4 (1.1–1.9)	0.003	1.6 (1.2–2.2)	**0.001**
30–39 years	137	66	(48)	1.2 (0.9–1.6)	0.179	1.4 (1.0–1.9)	0.038
Education level							
High education status	479	244	(51)	1		1	
Low education status	65	35	(54)	1.1 (0.8–1.3)	0.653	1.3 (1.0–1.7)	0.022
Designation of staff							
TLPMS (THN/THA/HA/LHV)	79	36	(46)	1		1	
Field supervisory staff (MW/PHS/VBDC staff)	465	243	(52)	1.1 (0.9–1.5)	0.295	0.9 (0.7–1.2)	0.580
Duty station							
Urban (Township hospital/MCH)	61	31	(51)	1			
Rural (station hospital/RHC/Sub-center)	483	248	(51)	1.0 (0.8–1.3)	0.939		
Initiation of elimination training in townships							
2016	179	69	(39)	1		1	
2017	365	210	(58)	1.5 (1.2–1.8)	< 0.001	1.5 (1.2–1.8)	**< 0.001**

*TLPMS* Township level program management staff, *THN* Township Health Nurse, *THA* Township Health Assistant, *HA* Health Assistant, *LHV* Lady Health Visitor, *MW* Midwife, *PHS* Public Health Supervisor, *VBDC* Vector-borne Diseases Control staff, *MCH* Maternal and Child Health Centre, *RHC* Rural Health Centre, *PR* Prevalence ratio

*Age information of 2 participants was missing, *n* = 542

### The qualitative strand

#### Training for malaria elimination

There were two levels of training programme for malaria elimination namely: training of trainers at the central level (Nay Pyi Taw) and training of field workers at township level. The training at the central level was conducted with the aid of an expert in the National Malaria Control Programme in 2016.

#### Perception towards training for malaria elimination

In order to improve the low level of knowledge of field management staff after exposure to the short training course, qualitative interviews further explored the perceptions of township level programme management staff. In this connection, key informants revealed their perceived needs for an improvement in the training programme in terms of the availability of supporting materials in electronic format. They expressed the need for more concise information as a necessity to include in future training, for instance how to complete the case investigation forms. This kind of updated information might improve knowledge and would support surveillance activities of field supervisory staff in line with malaria elimination guidelines.

Perceived needs for improvement in the training programme “It would be good if manuals are provided especially in a pdf format so that we can read these in our mobile phones. Should make accessible in any local websites in Myanmar language” TMO-KII

As might be expected, key informants identified the perceived readiness of trained health staff in terms of fieldwork, record keeping, and systematic reporting. In addition, some suggested case notifications should be channeled from the partner organizations including EHOs for accuracy in coverage with an emphasis on conflict-affected areas and areas with high population mobility. Key informants also perceived a low level of education of community members as one of the barriers that hampered public readiness in the elimination scenario apart from the unfavourable context.

“In conflict affected areas, I don’t think the NMCP can cover all activities needed for malaria elimination. In that case, we need assistance from the ethnic health organizations (EHO) and a direct contact with central VBDC. During collaboration with EHO, we train them for field work and reporting” (KII-THD).

“In areas of high population mobility, we establish check-in points of case investigation for migrant workers.” (KII-THD)

## Discussion

This is the first study conducted in Myanmar after the initiation of malaria elimination training in 2016–2017. This study addressed the essential elements in capacity building in a targeted malaria elimination scenario. Important findings included the knowledge gained by health staff at the township level after attending the malaria elimination training for 2 days coupled with perceived challenges and programmatic implications.

Even though the trainers used the National Malaria Elimination Guidelines as their core training tool, a significant impact on knowledge improvement after the training programme was not visible probably because of the short training period. Moreover, malaria case definitions were still not understood by the trained health staff and this might hamper their field surveillance activities. There was also a lack of proper understanding of what was meant by malaria elimination among 75% of respondents. These findings point to the need to plan properly for future refresher training which is consistent with the findings from other studies [10–12, 16].

The frontline health workers in younger age groups (18–29 years) pointed out the need for specific refresher training and supervision. The training programme for malaria elimination was not standardized in its early phase in each and every township in terms of training materials and provoking two-way interactive discussions. This might possibly explain why good knowledge was attained among those trained in 2016 compared to 2017. Whatever filling the training gaps can improve the implementation of desirable actions to further scale up malaria elimination in endemic regions [16]. Health workers in the periphery usually have less exposure to malaria cases in elimination settings and therefore they require more training on case detection and foci classification as has been observed in one recent study in China [17].

Challenges perceived for malaria elimination were distinct in conflict-affected areas as well as in areas of high population mobility and this might affect foci classification such as active foci, residual non-active foci and clear foci. Moreover, the provision of health education to community members could not be carried out adequately in these areas, as found in other studies in Myanmar and elsewhere [9, 10, 18]. The findings indicate a window of opportunity to strengthen the coordinated efforts between the NMCP and partner organizations for future training, refresher training and continuing technical support and to further promote community engagement in malaria elimination. Apart from that, preparing training materials in the local language including posters/flip charts with illustrations for malaria cases, category definition and workflow schema could be supportive. Complementing the method of text messaging to classroom teaching to train health workers for malaria elimination interventions improved healthcare performance in Uganda [19].

The conduct and report of this mixed-methods study followed STROBE guidelines [20] for observational studies in Epidemiology and COREQ guidelines for qualitative studies [21]. Study findings are generalizable to promote the scale-up of capacity building activities for malaria elimination in other developing countries taking into account the local operational context [22] as well as the availability of well-versed trainers, standardized training materials, methods and innovative approaches to fit into resource-constrained settings. However, by opting for a self-administered method in a quantitative survey, the reliability of responses for knowledge items was possibly in doubt.

The following programmatic implications should be taken into account. First, capacity building through training programmes for township level programme management staff and field supervisory staff must continue and be strengthened with oversight to ensure that early case finding, diagnosis and notification for appropriate treatment especially in remote sites are fully undertaken. Second, by using this evidence generated, the NMCP needs to arrange a thorough evaluation of elimination activities carried out by trained health staff together with voluntary health workers.

## Conclusion

The malaria elimination training programme in study townships was less effective in improving the knowledge of health staff for correct notification of malaria cases and treatment according to the National Malaria Elimination Guidelines. Apparently, healthcare staff did not have enough confidence to implement elimination activities for achievement of the elimination goal by 2020 especially in conflict-affected areas and in areas with high population mobility. In addition, regular monitoring and continuing guidance by the higher level management during field operations require attention. It is critical to develop and test alternative strategies for coordinated efforts with implementing partners to promote the scaling up of malaria elimination training programmes in targeted townships.

## Methods

### Study design

This study was a retrospective analysis of a sub-set of quantitative as well as qualitative data acquired through a mixed-methods explanatory sequential design [23] conducted between October 2017 and March 2018.

### Study setting

#### General setting

The Republic of the Union of Myanmar is administratively divided into 14 states/regions and Nay Pyi Taw Council Territory. Malaria is endemic in 291 out of 330 townships in Myanmar: 34 townships in the Yangon region and five townships in the Mandalay region (total 39 townships) were malaria-free in 2016 [24, 25].

#### Specific setting

As reported in the 2014 Myanmar National Census, the population in Yangon region (45 townships), Bago region (28 townships), Magway region (25 townships), Mandalay region (28 townships), Nay Pyi Taw Union Territory (8 townships) and Mon State (10 townships) was 7.4, 4.9, 3.9, 6.2, 1.2 and 2.1 million respectively. The annual parasite index (API) of the five regions except Mon State was less than 1 in 2016. In addition, there were conflict-affected townships in Bago region in which mobile migrant workers were also common as shown in published studies [9].

#### Performance of the National Malaria Control Program in malaria elimination

The NMCP is carrying out malaria control and elimination activities through BHS at the township level and involves Health Assistants (HA), Lady Health Visitors (LHV), midwives (MW), and Public Health Supervisors (PHS) who carry out other primary healthcare responsibilities. Under the supervision of BHS at rural health centers (RHC) and sub-centers (SC), village health volunteers (VHV) provide the malaria control services especially in hard-to-reach remote sites. In March 2016, the training of trainers (TOT) programme for malaria elimination was initiated at the central level led by the National malaria programme expert. The Standard Operational Procedures (SOP) for frontline health workers to carry out malaria elimination activities were not available at that time. Starting from 2017, malaria elimination trainings were extended to state/region and township level for malaria regional officers/team leaders in six states and regions where malaria elimination was targeted in 2020. During the 2-day training, knowledge on malaria elimination was imparted through lectures using powerpoint presentations and question and answer sessions.

### Sample size determination and sampling procedure

The quantitative strand: The original survey was conducted to outline the health system readiness in targeted malaria elimination settings in six out of 70 townships randomly selected from six states/regions of Myanmar which had already received a 2-days training for malaria elimination. The research team recruited a total of 544 respondents at the study sites including 11 VBDC staff and 533 BHS who were available at their health posts and gave consent to participate in the survey. This study extracted the responses of all participants from the original survey database.

The qualitative strand: One VBDC team leader/staff and one Head of the Township Health Department (THD) from each study site were purposefully selected for key informant interviews (KII). Except for two VBDC team leaders who were not available at the time of the survey, there was a total of 10 key informants in the original survey.

### Data collection, variables and data sources

#### The quantitative strand

For the quantitative survey, the research team members introduced the self-administered structured questionnaire to eligible respondents in each township. Eight out of 17 knowledge variables, inclusive of 20 sub items extracted from the original survey database, covered the comprehensive package of malaria elimination training guidelines: the definition of malaria elimination, the definition of a malaria case, activities for malaria elimination, the category of malaria cases, who should be tested for malaria diagnosis, types of foci investigation, core interventions for *Anopheles* vector control, and key messages about malaria elimination aimed at the community.

The remaining nine knowledge items and additional opinion items concerning 1-3-7 surveillance and response strategy were analysed separately for another manuscript that focused on health system readiness for malaria elimination.

#### The qualitative strand

Trained interviewers used the pretested guidelines for key informant interviews that focused on perceived needs, opinions and perceived readiness for malaria elimination by BHS and VBDC teams who worked for township level programme management. Perceived challenges and suggestions of frontline health workers in the early phase of malaria elimination were emphasized in another manuscript by triangulating with quantitative items as mentioned above, without any overlap with this study. The source of both quantitative data and qualitative data was the operational research/implementation research electronic database of the Department of Medical Research (DMR).

Operational definitions of key variables used in a quantitative survey are provided in Table 5.

**Table 5:** Operational definitions of key knowledge variables

Key variables	Operational definitions
Malaria case	Occurrence of malaria infection in a person in whom the presence of malaria parasites in the blood has been confirmed by a diagnostic test
Malaria elimination	Interruption of local transmission (reduction to zero incidence of indigenous cases) of a specified malaria parasite in a defined geographical area as a result of deliberate activities. Continued measures to prevent re-establishment of transmission are required.
Indigenous case	A case contracted locally with no evidence of importation and no direct link to transmission from an imported case
Introduced case	A case contracted locally, with strong epidemiological evidence linking it directly to a known imported case (first-generation local transmission)
Imported case	Malaria case or infection in which the infection was acquired outside the area in which it is diagnosed.
Relapse case	Malaria case attributed to activation of hypnozoites of *P. vivax* or *P. ovale* acquired previously.
Induced case	Other types of transmission (by blood transfusion, from mother to child transmission)
Recrudescent case	Recurrence of asexual parasitaemia of the same genotype(s) that caused the original illness, due to incomplete clearance of asexual parasites after antimalarial treatment.
Focus	A defined circumscribed area situated in a currently or formerly malarious area that contains the epidemiological and ecological factors necessary for malaria transmission Note: Foci can be classified as active, residual non-active or cleared.
Active foci	A focus with ongoing transmission.
Residual non-active foci	Transmission interrupted recently (1–3 years ago).
Cleared foci	A focus with no local transmission for more than 3 years and which is no longer considered residual non-active.

### Data analysis and statistics

The quantitative strand: Following double-entry using EpiData (version 3.1, EpiData Association, Odense, Denmark), the data were analysed by STATA (version 13.0, College Station, Texas USA). The knowledge score as a dependent variable was computed for eight key knowledge items concerning malaria elimination (inclusive of 31 sub-items) and each correct response was assigned a ‘1’ and incorrect and do not know was assigned a ‘0’. The total scores were further categorized as high or low based on the median value. Independent variables included age of the respondent, education level, the category of staff and the year of initiation of elimination training. The modified Poisson regression with robust variance estimates was performed to compute the adjusted prevalence ratio (APR) and 95% confidence intervals (CI) by incorporating all independent variables with a p value of 0.2 during bivariate analysis. The level of significance was set at p < 0.05. The STROBE guidelines for observational studies in epidemiology were used in the conduct and reporting of the quantitative study [20].

The qualitative strand: The transcripts in Myanmar language were coded and translated into English and a thematic analysis was done by ATLAS.ti 6.1.1. Two researchers performed a validity check on the coding. The COREQ guidelines were used for reporting the qualitative findings [21].

## Data Availability

Data are not available in public domain because they are currently being analysed in related papers. However, data are available with the corresponding author (ZNMH) and will be made accessible on reasonable request at the following e-mail: zarniminhein24@gmail.com.

## Abbreviations

APR: Adjusted prevalence ratio BHS Basic Health Staff CI Confidence intervals COREQ Consolidated Criteria for Reporting Qualitative Research DMR Department of Medical Research EHO Ethnic Health Organizations HA Health assistant IQR Interquartile range IRS Indoor residual spraying ITN Insecticide-treated nets KII Key Informant Interviews LHV Lady Health Visitor LLINS Long-lasting Insecticidal Nets MCH Maternal and Child Health Centre MW Midwife NMCP National Malaria Control Programme PHS Public Health Supervisor PR Prevalence Ratio RHC Rural Health Centre SC Sub-centers STROBE Strengthening the Reporting of Observational Studies in Epidemiology THA Township Health Assistant THN Township Health Nurse TLPMS Township level program management staff VBDC Vector-borne Diseases Control VHV Village Health Volunteers

## References

[1] World Health Organization (2017). Global Malaria Programme. A framework for malaria elimination.

[2] World Health Organization (2015). World Malaria Report 2015.

[3] World Health Organization (2015). Global Technical Strategy for Malaria 2016-2030.

[4] National Malaria Control Programme (2018). Malaria surveillance in elimination settings : an operational manual 2018 Version 1.0.

[5] Tun KM, Imwong M, Lwin KM, Win AA, Hlaing TM, Hlaing T (2015). Spread of artemisinin-resistant Plasmodium falciparum in Myanmar: a cross-sectional survey of the K13 molecular marker. Lancet Infect Dis.

[6] World Health Organization (2013). Emergency response to artemisinin resistance in the Greater Mekong subregion: regional framework for action 2013-2015.

[7] World Health Organization (2011). Global Plan for Artemisinin Resistance Containment (GPARC).

[8] Hlaing T, Wai KT, Oo T, Sint N, Min T, Myar S (2015). Mobility dynamics of migrant workers and their socio-behavioral parameters related to malaria in Tier II, Artemisinin Resistance Containment Zone, Myanmar. BMC Public Health.

[9] Win AYN, Maung TM, Wai KT, Oo T, Thi A, Tipmontree R (2017). Understanding malaria treatment-seeking preferences within the public sector amongst mobile/migrant workers in a malaria elimination scenario: a mixed-methods study. Malar J BioMed Central.

[10] Abeyasinghe RR, Galappaththy GNL, Gueye CS, Kahn JG, Feachem RGA. Malaria control and elimination in Sri Lanka: Documenting Progress and Success Factors in a Conflict Setting. 2012;7. 10.1371/journal.pone.0043162 PMC3430652 22952642

[11] National Malaria Control Programme. National Plan for Malaria Elimination (NPME) in Myanmar 2016-2030. Nay Pyi Taw, Myanmar: National Malaria Control Programme; 2016.

[12] World Health Organization (WHO). Service Availability and Readiness Assessment (SARA); An annual monitoring system for service delivery. Implementation guide. Serv. Availab. Readiness Assess. (SARA); An Annu. Monit. Syst. Serv. Deliv. Implement. Guid. Geneva, Switzerland; 2015.

[13] Hlongwana KW, Tsoka-Gwegweni J. Towards the implementation of malaria elimination policy in South Africa: The stakeholders’ perspectives. Glob Health Action Taylor & Francis. 2017;10. 10.1080/16549716.2017.1288954 PMC5496171 28475435

[14] Lee EH, Olsen CH, Koehlmoos T, Masuoka P, Stewart A, Bennett JW (2017). A cross-sectional study of malaria endemicity and health system readiness to deliver services in Kenya, Namibia and Senegal. Health Policy Plan.

[15] Lu G, Liu Y, Wang J, Li X, Liu X, Beiersmann C (2018). Malaria training for community health workers in the setting of elimination: a qualitative study from China. Malar J BioMed Central.

[16] Wirth DF, Casamitjana N, Tanner M, Reich MR (2018). Global action for training in malaria elimination. Malar J.

[17] Wang R, Tang S, Yang J, Shao T, Shao P, Liu C, et al. Improving local health workers’ knowledge of malaria in the elimination phase—determinants and strategies: a cross-sectional study in rural China. Malar J. 2017. 10.1186/s12936-017-1865-1 PMC5438496 28526083

[18] Han KT, Wai KT, Oo T, Thi A, Han Z, Kyin D, et al. Access to primaquine in the last mile: challenges at the service delivery points in pre-elimination era , Myanmar Tropical Medicine and Health; 2018;10–3. 10.1186/s41182-018-0115-8 PMC6145114 30250397

[19] Rassi C, Gore-Langton GR, Walimbwa BG, Strachan CE, King R, Basharat S, et al. Improving health worker performance through text messaging: a mixed-methods evaluation of a pilot intervention designed to increase coverage of intermittent preventive treatment of malaria in pregnancy in West Nile, Uganda. PLoS One Public Library of Science. 2018;13. 10.1371/journal.pone.0203554 PMC6126848 30188956

[20] Von Elm E, Altman DG, Egger M, Pocock SJ, Gøtzsche PC, Vandenbroucke JP (2014). The Strengthening the Reporting of Observational Studies in Epidemiology (STROBE) Statement: guidelines for reporting observational studies. Int J Surg.

[21] Tong A, Sainsbury P, Craig J (2007). Consolidated criteria for reporting qualitative research(COREQ): A 32-item checklist for interviews and focus groups. Int J Qual Health Care.

[22] Hlongwana KW, Sartorius B, Tsoka-Gwegweni J (2018). Malaria programme personnel’s experiences, perceived barriers and facilitators to implementing malaria elimination strategy in South Africa. Malar J BioMed Central.

[23] Fetters MD, Curry LA, Creswell JW (2013). Achieving integration in mixed methods designs—Principles and practices. Health Serv Res.

[24] Ministry of Health and Sports. Health in Myanmar 2014. Nay Pyi Taw: Ministry of Health and Sports; 2015. 2014. p. 1–142. http://www.moh.gov.mm/fle/managinghealthworkforce.pdf. Accessed 14 Jun 2019

[25] Vector Borne Disease Control Programme (2017). VBDC Annual Report 2016.

